# Hybridoma technology; advancements, clinical significance, and future aspects

**DOI:** 10.1186/s43141-021-00264-6

**Published:** 2021-10-18

**Authors:** Sanchita Mitra, Pushpa Chaudhary Tomar

**Affiliations:** Department of Biotechnology, Faculty of Engineering & Technology, Manav Rachna International Institute of Research and Studies, Faridabad, Haryana 121004 India

**Keywords:** Monoclonal antibodies, Hybridomas, Chimeric, Therapeutic, Cryopreservation

## Abstract

**Background:**

Hybridoma technology is one of the most common methods used to produce monoclonal antibodies. In this process, antibody-producing B lymphocytes are isolated from mice after immunizing the mice with specific antigen and are fused with immortal myeloma cell lines to form hybrid cells, called hybridoma cell lines. These hybridoma cells are cultured in a lab to produce monoclonal antibodies, against a specific antigen. This can be achieved by an in vivo or an in vitro method. It is preferred above all the available methods to produce monoclonal antibodies because antibodies thus produced are of high purity and are highly sensitive and specific.

**Main body of the abstract:**

Monoclonal antibodies are useful in diagnostic, imaging, and therapeutic purposes and have a very high clinical significance. Once hybridoma cells become stable, these cell lines offer limitless production of homogenized antibodies. This method is also cost-effective. The antibodies produced by this method are highly sensitive and specific to the targeted antigen. It is an important tool used in various fields of research such as in toxicology, animal biotechnology, medicine, pharmacology, cell, and molecular biology. Monoclonal antibodies are used extensively in the diagnosis and therapeutic applications. Radiolabeled monoclonal antibodies are used as probes to detect tumor antigens in the living system; also radioisotope coupled antibodies are used for therapeutic target specific action on oncogenic cells.

**Short conclusion:**

Presently, the monoclonal antibodies used are either raised in mice or rats; this poses a risk of disease transfer from mice to humans. There is no guarantee that antibodies thus created are entirely virus-free, despite the purification process. Also, there are some immunogenic responses observed against the antibodies of mice origin. Technologically advanced techniques such as genetic engineering helped in reducing some of these limitations. Advanced methods are under development to make lab-produced monoclonal antibodies as human as possible. This review discusses the advantages and challenges associated with monoclonal antibody production, also enlightens the advancement, clinical significance, and future aspects of this technique.

## Background

**Fig. 1:**
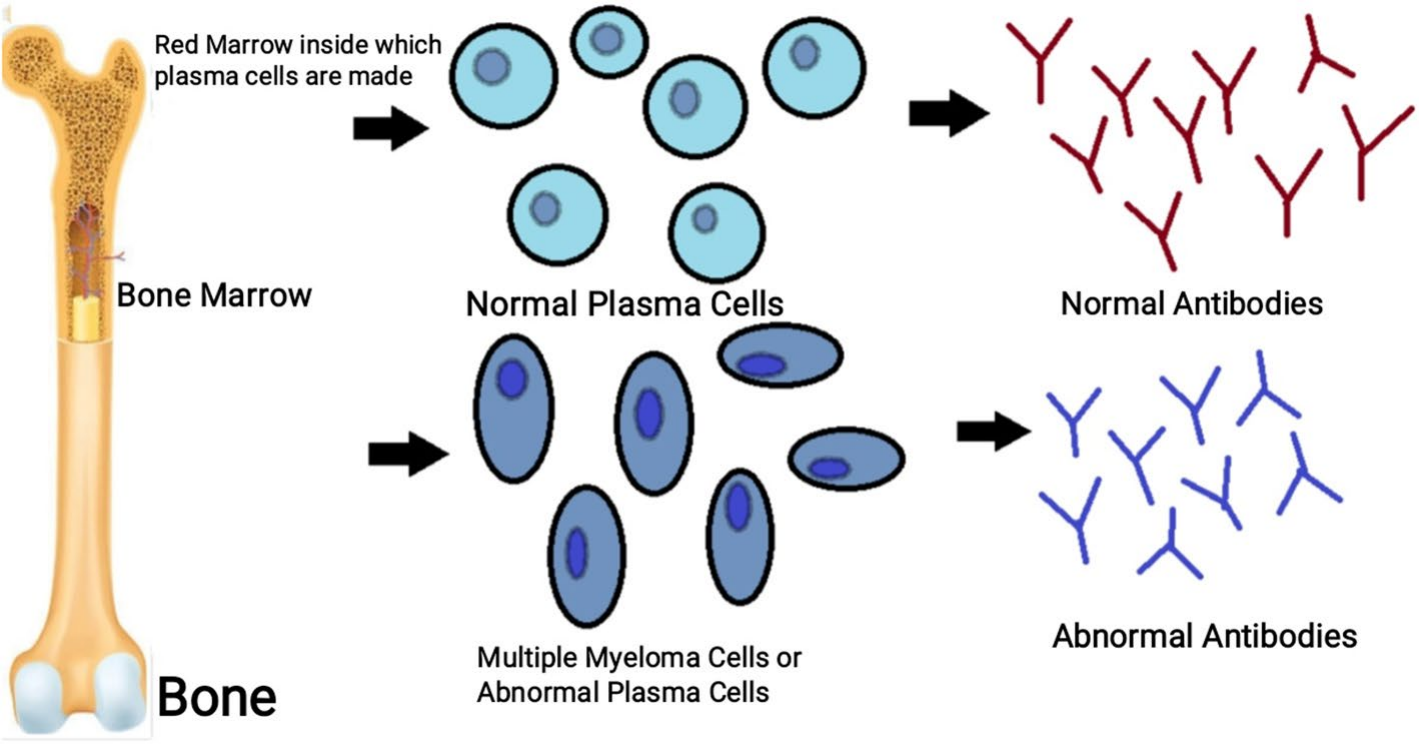
Formation of myeloma cells

Antibodies are mainly produced for diagnostic and therapeutic applications. Monoclonal antibodies were discovered in 1975. In 1975, Kohler and Milstein discovered a technique called hybridoma technology for the production of monoclonal antibodies. It is one of the most widely used techniques in modern research and studies [1]. Kohler and Milstein [2] developed a system in which antibody-producing B cells were fused with immortal cancerous cell lines such as myeloma cells [3] (Fig. 1), creating an immortal hybrid cell line that produces antibodies limitlessly.

Hybridoma technology produces monoclonal antibodies (mAbs) specific to antigens. These cell lines can also be cryopreserved for a long period of time. Hybridoma technology has resulted in the production of a variety of different monoclonal antibodies with specificity for a specific antigen. Antigen molecules include enzymes, hormones, internal and external structures of bacteria, viruses, and eukaryotic cells. Monoclonal antibodies produced by this method are highly specific antibodies, which are derived from a single parental B cell clone [4].

This discovery is considered one of the most important turning points in the field of biotechnology. Hybridoma technology has expanded the discovery and production of antibodies for multiple applications [1]. Researchers mostly prefer hybridoma technology, for monoclonal antibody production over other methods to maintain a convenient, cost-effective, and limitless production of monoclonal antibodies [5].

**Fig. 2:**
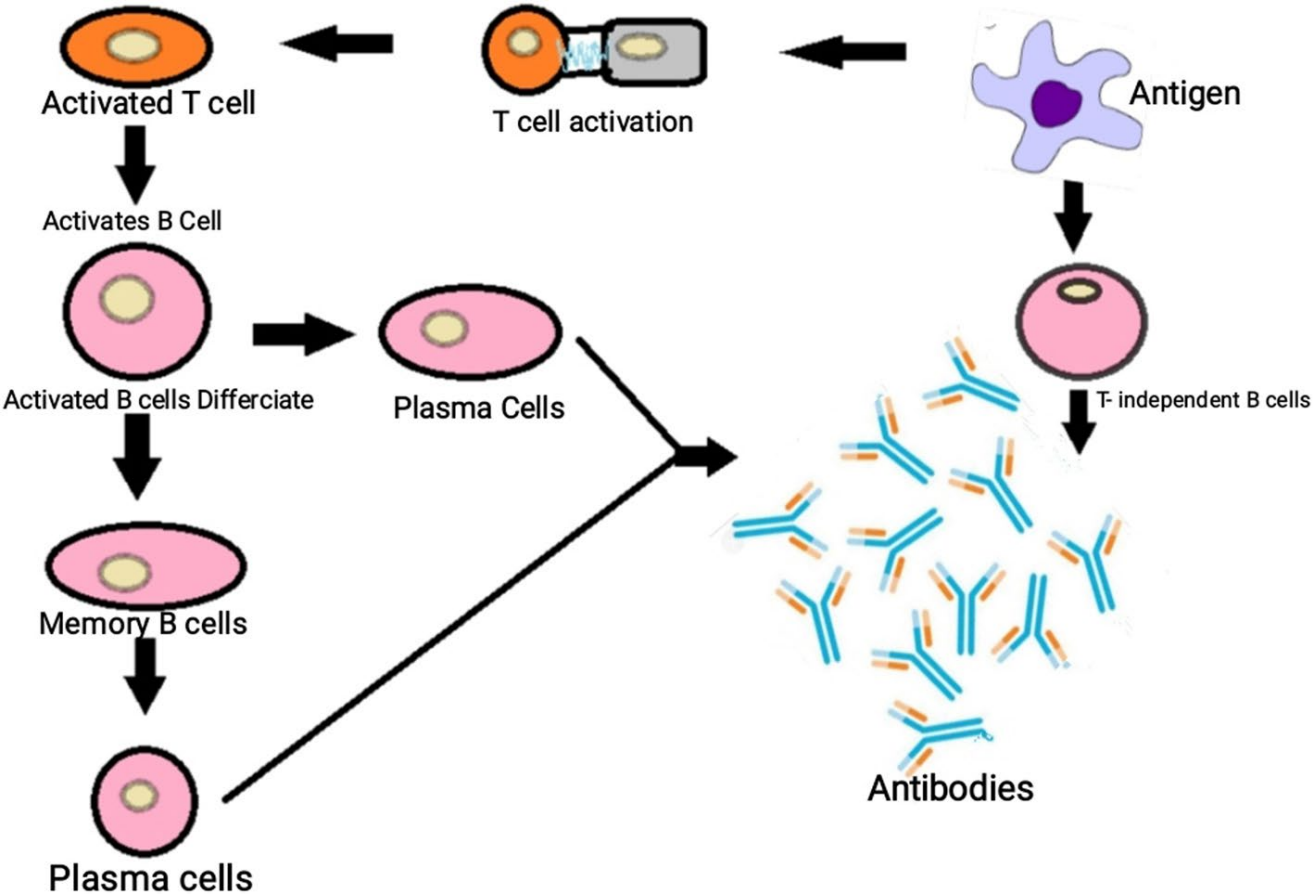
Activation of B cells and production of antibodies

Antibodies are glycoproteins produced by the B lymphocytes (Fig. 2). These antibodies are referred to as immunoglobulins. Antibodies are heterodimers and are created from 2 structural units, a heavy chain, and a light- chain. The N-terminal end, with approximately one hundred ten amino acids of the light and heavy chains, is referred to as variable regions. It is crucial for antigen recognition. The glycoproteins are classified into five different types on the basis of heavy chains which are in turn based on the structure of crystallizable fragments (Fc) that are linked to antigen-binding fragments. Antibodies are classified into five distinct isotypes based on differences in Fc regions, IgE, IgA, IgD, IgG, and IgM [6].

Antibodies are divided into two types based on their origin from lymphocytes: monoclonal antibodies and polyclonal antibodies. Each polyclonal and monoclonal antibody has advantages and disadvantages that make them equally suitable for various applications.

### Polyclonal antibodies

Polyclonal antibodies (pAbs) are immunoglobulin molecules created by totally different B cell lineages and these polyclonal antibodies react against multiple epitopes of a particular antigen. Polyclonal antibodies are heterogeneous mixtures of various antibodies with the potential to identify multiple epitopes. Polyclonal production of antibodies will increase the potency of the immune system and offer it the potential to convey a fast and effective response against any bacterial or viral insult. Polyclonal sera have multiple epitope binding properties, which makes them a useful chemical agent for a wide range of applications. Polyclonal sera have been used for many years as a treatment against toxin-producing microorganisms as well as disease-specific microorganisms [6]. The method of manufacturing leads to batch-to-batch variability that poses a risk of transmitting blood-borne diseases. Another disadvantage is that these antibodies have low specificity, and hence, comparatively high doses are needed to achieve the desired result during experimentation. Also, polyclonal antibodies cannot be used to treat chronic diseases. Due to a number of these disadvantages associated with polycomb antibodies, there is a necessity for isolating antibodies that are specific. Therefore, mAbs are created by somatic cell technology, which is sensitive, highly specific [6].

### Monoclonal antibodies

Monoclonal antibodies are a homogeneous mixture of antibodies that are monospecific in nature. These antibodies have affinity and specificity towards one epitope of a selected antigen (monovalent affinity). In the event of the development of monovalent antibodies, the scope of therapeutic and diagnostic applications has expanded encompassing various fields of biotechnology such as molecular biology, toxicology, biochemistry, and medicine. Out of the several techniques developed over years to produce monoclonal antibodies (single lymph cell amplification or by culturing strategies), hybridoma technology is one of the most important and most commonly used [7].

## Main text

### Preparation of monoclonal antibodies using hybridoma technology

#### Immunization

The first step involves injecting the laboratory animals like rabbits or mice with a selected antigen against which the antibodies are raised through a series of injections over a period of several weeks to stimulate B cell differentiation into plasma B cells and memory B cells. Once a sufficient number of antibodies are created in the animal serum following a few weeks of immunization, the animal is sacrificed [8].

#### Isolation of B lymphocytes

Following sacrifice, the spleen is removed in aseptic conditions to isolate the activated B-cells. This procedure is performed using density gradient centrifugation. The presence of antibodies in the Serum is identified using methods like ELISA [8] or flow cytometry. The serum contains the activated B lymphocytes (that produce antibodies). The activated B lymphocytes are then fused with myeloma cells.

#### Preparation of myeloma cell lines

Few weeks before the cell fusion, metastatic tumor cells are incubated in 8-azaguanine to get non-functional hypoxanthine-guanine phosphoribosyltransferase (HGPRT) genes in the myeloma cells. Non-functional HGPRT can stop the assembly of nucleotides from the salvage pathway and makes the metastatic tumor cells sensitive to HAT media as the preferred method in hybridoma technology [8].

#### Cell fusion

Cell fusion is the process in which the activated B lymphocytes are fused with HAT-sensitive myeloma cells. This step is performed by centrifugation of freshly obtained activated B-cells with HAT-sensitive myeloma cells in a fusion-promoting media. Polyethylene glycol (PEG) is used in this procedure [8]. PEG helps in the fusion of cells by promoting the fusion of the plasma membrane of the myeloma cells with the plasma membrane of the antibody-producing cells, thus giving rise to a cell with more than one nucleus, forming heterokaryon. Another method used for fusion is electrofusion, in which cells are fused under the effect of an electric field. This method is more efficient than the previous method [8, 9].

#### Hybridoma selection

In the PEG-containing media, cells are fused to form hybridoma cells but even the most efficient fusion method will allow the formation of only about 1 to 2% of fused hybridoma cells. Furthermore, about 1 in 100 cells will be viable hybrid cells. Therefore, there are a number of unfused cells within the media [1]. This step allows the selection of the fused cells from all the unfused cells. This is achieved by incubating the cell mixture followed by culturing for 10–14 days in HAT media (a selection media). HAT medium contains hypoxanthine-aminopterin-thymidine. Aminopterin present in HAT media blocks the power of cells to synthesize nucleotides by the de novo synthesis pathway. Hypoxanthine and deoxythymidine allow cells with functional hypoxanthine-guanine phosphoribosyltransferase (HGPRT) genes to survive through salvage pathways. Due to a limited life span, unfused B cells perish within a few days. Unfused malignant neoplastic cells die as a result of the lack of the hypoxanthine-guanine phosphoribosyltransferase (HGPRT) gene. The presence of aminopterin blocks their ability to synthesize nucleotides through the de novo pathway [10]. Therefore, the remaining viable cells left in the media are the hybrid cells; these hybrid cells have the ability to grow and divide on HAT media because they have functional HGPRT gene from the B lymphocytes, which make them HGPRT positive, and thus, they can grow in unlimited concentration on HAT media [8].

#### Screening of hybridoma cells

HAT-selection hybridoma cells are transferred to ELISA plates, where each well houses a single hybridoma cell. This is achieved using the limiting dilution method [8]. The genes of the B cell lineage present in the hybridoma cells produce a specific antibody with a specific epitope; this antibody is known as “monoclonal antibody.” There may be other hybridomas present in other wells producing antibodies specific to another epitope for the same antigen. After the separation and isolation of different hybridomas, screening is performed for selecting hybridomas that produce the desired antibodies targeting specific epitopes for an antigen [5].

#### Cloning and propagation of hybridoma cell

Hybridomas producing desired antibodies are selected and are then transferred into large culture vessels or flasks; the hybridoma cell lines are cultured using in vivo *or* in vitro methods. These hybridoma cells can be maintained and preserved in the culture media for the production of monoclonal antibodies [5].

#### In vivo

The in vivo method uses mice for the production of monoclonal antibodies. Mice are injected intraperitoneally with 105 to 110 viable hybridoma cells. After a few weeks, the ascites fluid is collected from an anesthetized mouse. Ascites fluids are contaminated with mouse immunoglobulins to some extent and isolation of monoclonal antibodies requires purification If purity of the antibody is important, then this technique may be inconvenient [2, 5].

#### In vitro

**Fig. 3:**
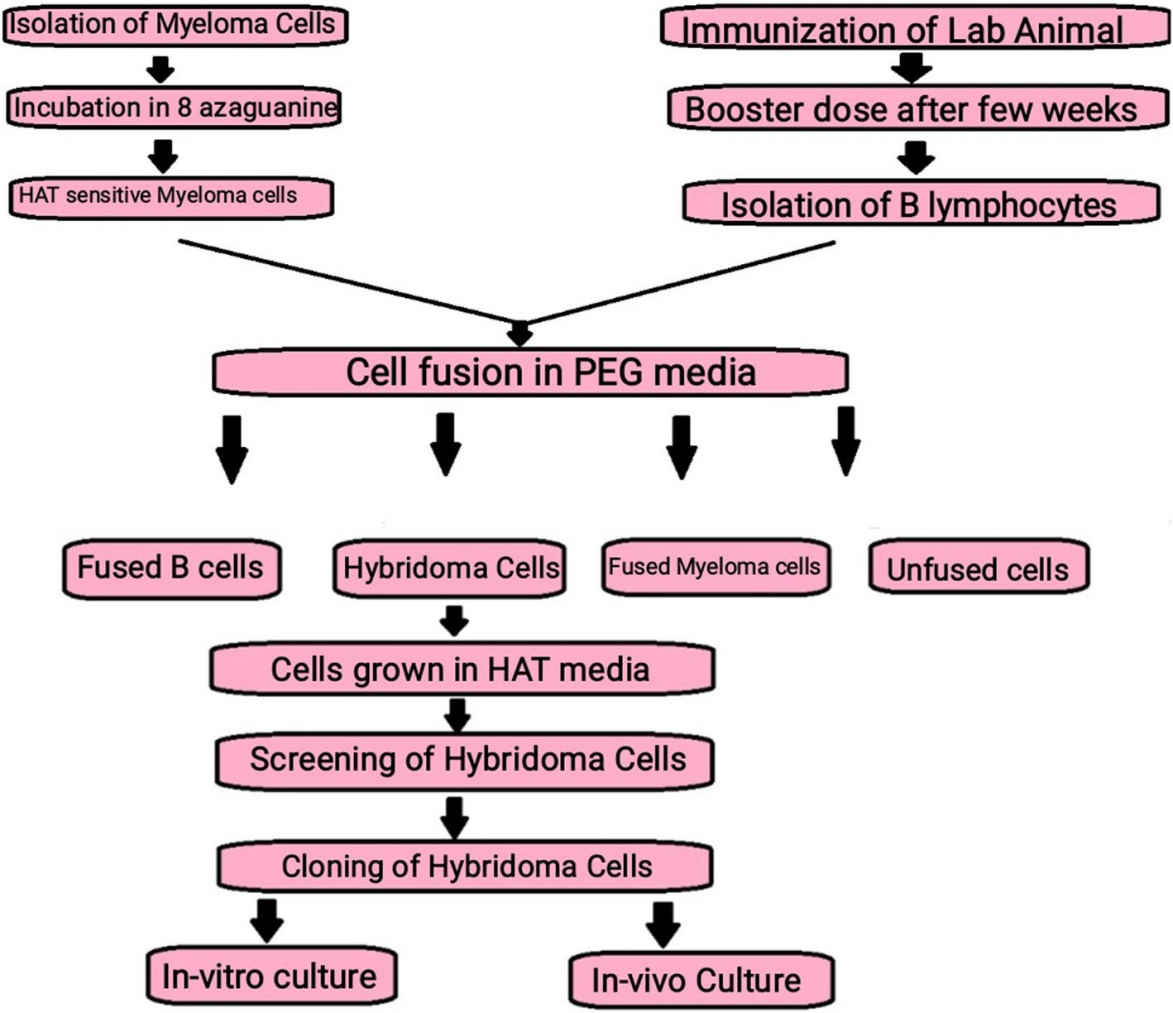
Flow chart showing the methodology of hybridoma technology

**Fig. 4:**
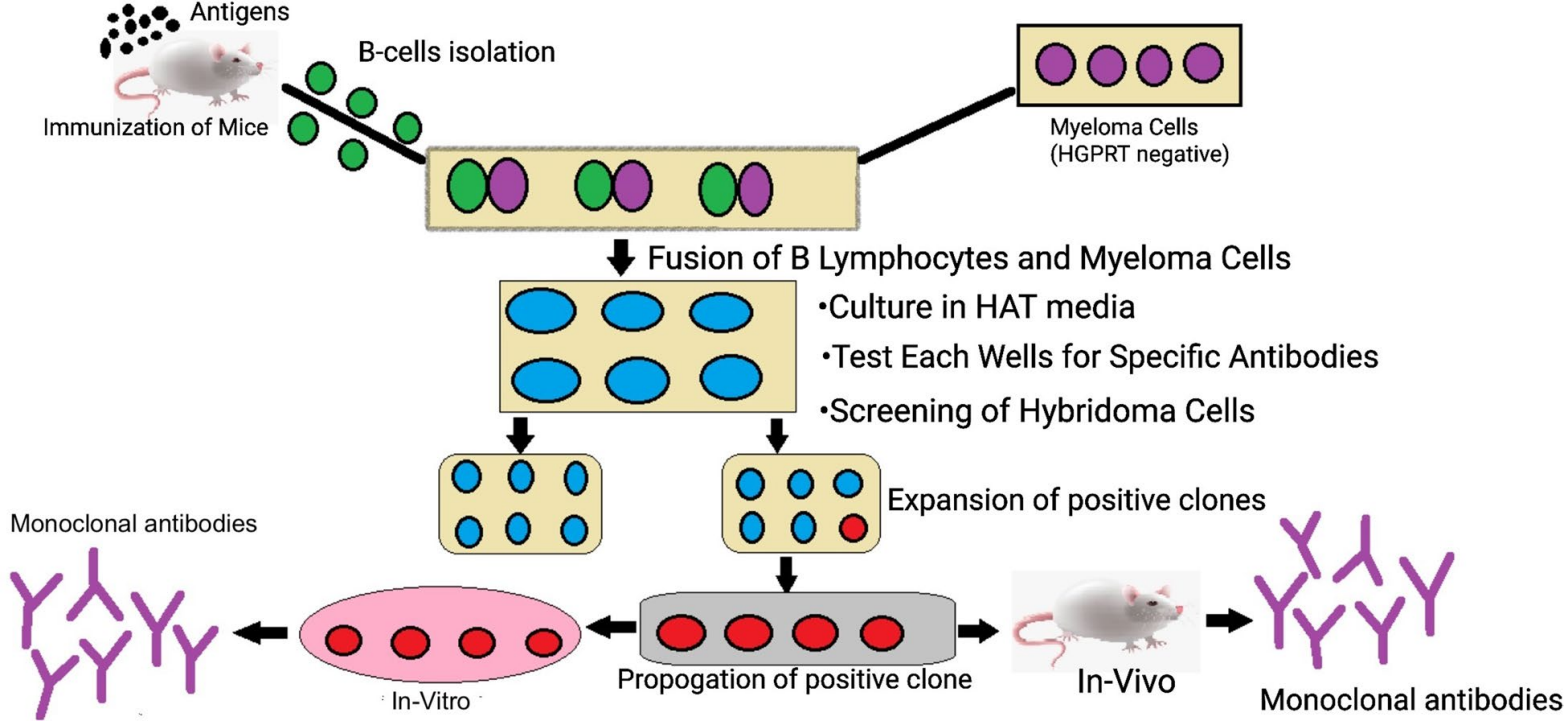
Diagram showing the production of monoclonal antibodies

This is another method in which hybridoma cells are cultured in laboratory conditions. It involves growing the hybrid cells in a culture media followed by isolation of monoclonal antibodies from the media. This method is more suitable for the culturing of hybrid cells as it reduces the risk of contamination. In vitro antibody production leads to the production of highly pure antibodies [5, 11] (Figs. 3 and 4).

### Clinical significance of hybridoma technology

**Fig. 5:**
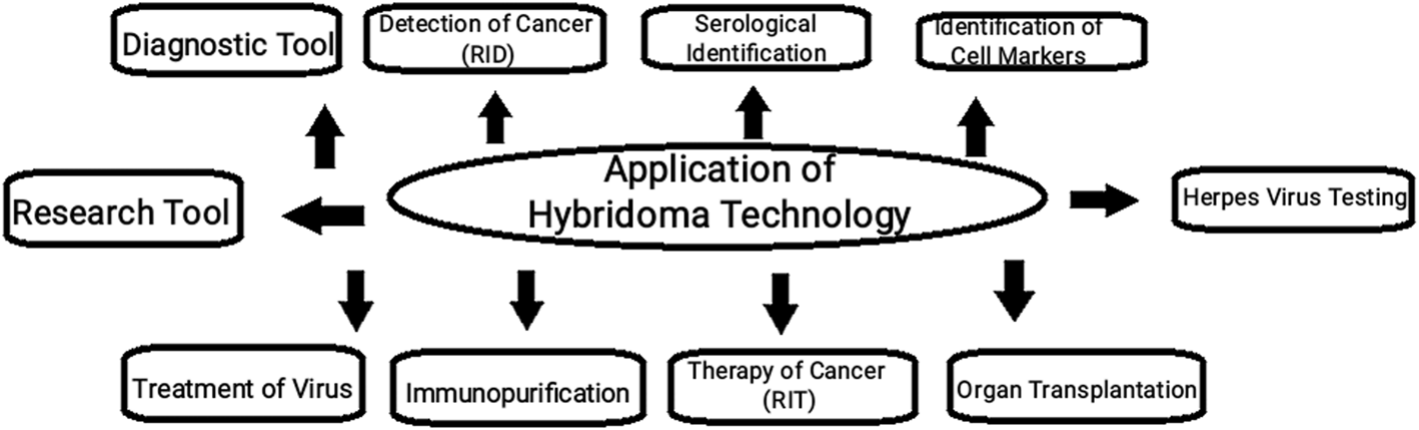
Application of hybridoma technology

Hybridoma technology, as mentioned earlier, is one of the most common methods for producing monoclonal antibodies. The applications of monoclonal antibodies are diverse and these include (Fig. 5):

#### Diagnostic testing

Monoclonal antibodies are commonly employed in the diagnosis of a variety of disorders. It is used to check the presence of any foreign antigen such as toxins, drugs, hormones, or internal and surface proteins of bacteria or viruses [12].

#### Testing of pregnancy

Monoclonal antibodies are used to identify the presence of human chorionic gonadotropin [hCG] as a mark for recognition of pregnancy [8].

#### Radioimmunodetection (RID) of cancer

Monoclonal antibodies are also used to detect the presence of specific tumor-type in the body. In this technology, antibodies are labeled with radioactive tags to check the presence of any type of carcinomas or cancer-specific cells in the body [13].

#### Malaria herpes virus testing

mAbs are used in the diagnosis of various diseases caused by viruses such as malaria herpes viruses [5].

#### Identification of different strains of pathogens

Monoclonal antibodies can be used to differentiate between different strains of a single pathogen, for example, *Neisseria gonorrhoeae* [5]*.*

#### Serological identification of ABO blood groups

Monoclonal antibodies can also be used in the serological identification of blood groups. The antibodies can be isolated from the sera of a person stimulated by the A or B blood group [10].

#### Radioimmunotherapy (RIT) of cancer

This technique is similar to RID; in RIT, monoclonal antibodies are used to target tumor cells; following this, the targeted cells are then killed using a lethal dose of radiation; the advantage of this technique is that it minimizes the radiation exposure by other normal body cells [13, 14].

#### Cancer treatments through drugs

Monoclonal antibodies are also used in targeted chemotherapy. A drug named rituximab, sold under the brand name of Rituxan, was approved by FDA for commercial use, for the targeted treatment of cancers mainly lymphomas [7, 15].

#### Viral disease treatment

Monoclonal antibodies are also being tested in treatments of previously incurable diseases such as AIDS [2].

#### Specific cell identification and their functions

Monoclonal antibodies can be used to identify and monitor certain cell populations or even molecules in a living system.

#### Organ transplantation

Monoclonal antibodies are used for the inactivation of T-lymphocytes that play a role in the rejection of transplanted organs. Monoclonal antibodies such as OKT3 play an important role by interfering with T-cell function in graft rejection. It is a monoclonal antibody that targets the CD3 receptor, a membrane protein found on T cell surfaces [2, 5].

#### Rhesus disease immunization

The UK Blood Products laboratory has been working on research to develop a possibility of substituting mAb rhesus immunization and replacing it with serum [16].

#### Immunopurification

This technique has been used in the purification of individual interferons and can be used in the purification of proteins and enzymes.

### Advantages of hybridoma technology

- ■ Hybridoma technology produces highly pure and specific antibodies (monoclonal antibodies).
- ■ This method is highly reproducible and scalable [1].
- ■ This method provides an unlimited production of monoclonal antibodies.
- ■ It can be used to perform highly sensitive and specific assays.
- ■ There is no need to maintain the animal in the laboratory for the production of antibodies [in vitro method] [11].
- ■ In this method, the purity of antigen or immunogen is not a prerequisite.
- ■ The selection method is useful in the identification of the right clones against a specific antigen.
- ■ In vivo production of antibodies ensures the formation of a mixture of variable and constant domains. The generated antibodies have a high affinity towards the epitope of a targeted substance [6].
- ■ This method is not labor-intensive; in vitro antibody generation techniques require the use of immune libraries
- ■ Antibody reliability is vital to each analysis and assay development and is one of the key features of hybridoma technology. Once hybridoma cells become stable, these offer limitless production of cost-effective, homogenized antibodies [1].
- ■ The perfect tool for research, in various fields such as toxicology, animal biotechnology, medicine, pharmacology, etc.
- ■ Monoclonal antibodies are widely employed in diagnostic and therapeutic procedures [17].
- ■ It is used in various chemotherapeutic regimens to treat various cancer types.
- ■ Used widely in research for the production of vaccines.

### Challenges of hybridoma technology

- ■ This is a time-consuming method, requiring 6 to 9 months.
- ■ The method is quite expensive and requires considerable effort in production.
- ■ This method is not suitable for producing antibodies against small peptides and fragment antigens.
- ■ Hybridoma culture suffers from a high risk of contamination [10, 18].
- ■ This system of antibody production is now developed for only mice and rats and researchers are working continuously to develop antibodies of human origin [19].
- ■ The viable efficiency of cells is quite low. More than 99% of the cells die during the process of cell fusion reducing the efficiency of the method and also reducing the variety of helpful antibodies which will be made against a specific substance.
- ■ If monoclonal antibodies are generated against a single antigenic determinant, they do not show cross-reactivity with other antigenic determinants. Retroviruses are a common incidence within mammalian chromosomes. Generally, animals like mice that are used in the production of monoclonal antibodies could carry many viruses such as viscus virus, retrovirus, reovirus, herpes virus, and thymic virus, leading to cross-contamination or infection in humans [18]. This poses a major threat for cross disease transfer from mice or rats to the human.
- ■ Despite purification, there is no guarantee that monoclonal antibodies made using the hybridoma technique are virus-free.
- ■ The fusion of human lymphocyte and mouse myeloma cells may result in the production of unstable fused cells.
- ■ In humans, there are no stable myeloma cells, suitable for the process of antibody production that can be used to substitute mouse myeloma cells.

### Advancement and future aspect in hybridoma technology

Hybridoma technology has brought a revolution in cell biology, immunology, biotechnology, toxicology, pharmaceutical research, and medical research. Prior to the introduction of hybridoma technology, antibodies were created by immunizing laboratory animals followed by isolation of the sera, which may then be put to therapeutic use. But this method has many disadvantages such as the occurrence of allergic and hypersensitive reactions in the patients. Furthermore, there were no clinical trials for the administration of the crude sera. Hybridoma technology brought a paradigm shift as it led to the production of highly specific and sensitive monoclonal antibodies in huge amounts presently [17]. The mAbs have been used extensively in diagnostics also including the use in the detection of cancer. These are used extensively in cancer therapy as well. However, this method also has some limitations such as immunogenic responses but these limitations are rare and could be overcome with advanced study and research in this field [20, 21]. Technologically advanced techniques such as genetic engineering helped in reducing immunogenicity.

**Fig. 6:**
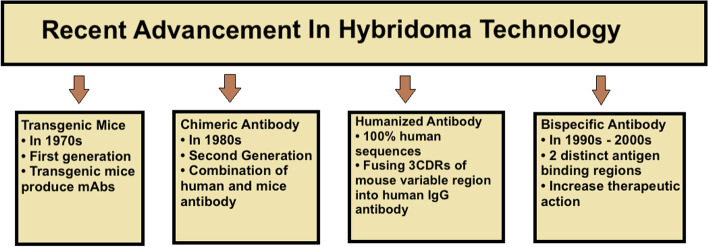
Advancement in the field of hybridoma technology

Advancement in the field of hybridoma technology (Fig. 6):

1. *Transgenic mice (first generation, the 1970s)*: The first generation of transgenic mice protein features two identical heavyweight chains and two identical lightweight chains (CH, CL), as well as variable domains (VH, VL). This protein also contains associated antigen-binding sites (CDRs), and immunogenicity is induced by the constant (Fc) region of the associate protein [22].
2. *Chimeric antibody (second generation, the 1980s)*: Chimeric monoclonal antibodies are created by substituting a consistent segment of human IgG protein for the constant segment of mouse protein [23–26].
3. *Humanized antibody*: More than 90% of human sequences are present in the humanized protein. Human immunoglobulin G antibodies are completely humanized by fusing the DNA of three CDRs from the mouse variable regions into human immunoglobulin G antibodies [27]. These totally humanized antibodies are made with the help of transgenic mice that have human immunoglobulin; therefore, they include 100% human sequences and are needed in fewer quantities than the antibodies of mice origin [9, 23, 28].
4. *A bispecific antibody (third generation, the 1990s, and 2000s)* aids in the prolongation of the half-life of monoclonal antibodies and the enhancement of their therapeutic activities. Bispecific antibodies are created from 2 distinct antigen-binding regions, and thus, they can bind to two totally different antigens [29].

The Food and Drug Administration (FDA) of the United States of America and the European Union Health Authorities have proprietary rights to a wide range of monoclonal antibodies for therapeutic application. These antibodies are effective against a variety of lethal disorders, including autoimmunity, inflammatory reactions, surgical organ rejection, hematological malignancies, and other human cancers [30]. Several monoclonal antibodies are now at various stages of clinical trials against lethal illnesses such as HIV, AIDS, and Ebola [10].

Presently, only monoclonal antibodies of mouse origin are being used, which sometimes triggers immunological responses (including allergies, etc.) [30].

### FDA-approved monoclonal antibodies

FDA has approved many monoclonal antibodies to be available commercially [31].

- *Margetuximab*, a drug that is used in conjunction with chemotherapy to treat patients with HER2-positive metastatic breast cancer, was approved in the year 2020 [15].
- *Evinacumab* is used in conjunction with other medications to decrease cholesterol in adults and children aged 12 and above, who have a hereditary form of high cholesterol [15].
- *Dostarlimab* is used to treat individuals with endometrial cancer that has progressed or relapsed following treatment with previous chemotherapeutic drugs [15].
- *Satralizumab* is used in the treatment of neuromyelitis optica spectrum disease (NMOSD) which is a rare, chronic autoimmune illness that affects the central nervous system and causes inflammation of the optic nerves, spinal cord, or brain [15, 32].
- *Isatuximab* is used in the treatment of multiple myeloma [15, 33].
- *Crizanlizumab* is used to ease severe pain caused in patients with sickle cell anemia [15, 32, 33].
- *Ibalizumab* helps manage HIV infection; this medicine is used with other HIV drugs. It aids in the reduction of HIV in the body, allowing the immune system to function more effectively [32, 33].
- *Benralizumab* is used in adults and children (12 years of age and above?) in combination with other medicines to prevent chest tightness, wheezing, trouble breathing, and cough caused by asthma [33].
- *Atoltivimab, maftivimab, and odesivimab-ebgn* are FDA-approved mAbs used for the treatment of Zaire ebolavirus. It comprises a fixed-dose combination of three monoclonal antibodies [34].
- *Aducanumab i*s a drug used in the treatment of Alzheimer’s disease (AD). It is an amyloid beta-directed monoclonal antibody that works to decrease the development of aggregated forms of Amyloid-beta (A) in the brains of patients suffering from Alzheimer’s disease [15].
- *Eptinezumab* is a medicine intended to assist people with migraines. It is a monoclonal antibody that targets the alpha and beta forms of calcitonin gene-related peptides (CGRP) in the brain [15, 33].
- *Brolucizumab* is used to treat severe eye disease (wet age-related macular degeneration). This medicine can aid in the preservation of eyesight and the prevention of blindness [15].
- *Risankizumab* is used in the treatment of plaque psoriasis [15, 32, 33].
- *Romosozumab* is used to treat bone density loss (osteoporosis) in post-menopausal women who are at a high risk of bone fractures. It works by boosting bone strength and density [15, 33, 35].

### Advance techniques to produce humanized monoclonal antibodies

#### Transformation of human B-lymphocytes by viral technique

B-cells are antibody synthesizing cells within the body; however, B cells cannot grow on their own within the culture media; therefore, this limitation is overcome by the infectious transformation of B cells with viruses [36] such as Epstein Barr virus (EBV) [37, 38]. EBV-mediated remodeling of B cells allows them to grow in culture media and, henceforth, can be used to manufacture monoclonal antibodies [39]. However, the yield of mAbs is incredibly low. Production of human mAbs is done using severe combined immunodeficiency disease mouse. The mouse stricken by severe combined immunological disorder (SCID) sickness does not have a functional immune system. These varieties of mice are utilized by immunizing them with particular antigens to supply human mAbs [40].

#### Genetically modified laboratory animals to produce human MAbs

In the past many decades’ several tries have been created to introduce human immune globulin genes into the ordination of the mice, to develop a genetically changed animal with human immune globulin genes. These transgenic mice have the potential to provide human immunoglobulins once vaccinated with a specific antigen. The B cells made by these transgenic mice are used to produce monoclonal antibodies. In the higher than approaches, the sole limitation is that the yield of mAbs is incredibly low. Researchers continue their work to go looking for higher and better alternatives [9, 24].

#### Production of human-mouse MAbs using genetic engineering

With the advancement within the field of gene-splicing, presently it is possible to feature bound human factor segments to a mouse order. The antibodies created by this technique are referred to as humanized antibodies. In this technique, the Fv region of human immune globulin is substituted by the mouse Fv region. The polymer sequence of the Fv sections of each L and H chain of human antibodies is replaced with the polymer sequence of the Fv regions of a mouse antibody. The newly developed humanized being antibodies have the Fc region of human immune globulin [7]. This technique stimulates specific immunologic responses against a particular antigen. These humanized antibodies are found to be effective against neoplasm cells *In-vitro*.

#### Substituting human Ig with mouse CDRs

In this human, mAbs are successfully developed that contain mouse complementary deciding regions (CDRs) [19]. During this methodology, the CDRs genes of humans are substituted by the genes of a mouse. These chimeral antibodies function as a good therapeutic agent. Presently several monoclonal antibodies are developed victimizing this system [25]. However, this system holds an obstacle. This methodology is kind of overpriced and long.

#### Production of Bi-specific monoclonal antibodies

In this technique, the arms of fab (antigen-binding arms) have 2 completely different specificities for 2 different epitopes of two different antigens; these forms of antibodies are referred to as Bi-specific monoclonal antibodies. These are generated either by fusing 2 completely different hybridomas or by recombinant DNA technology. These antibodies are helpful in the treatment of various diseases [29, 41].

#### Production of monoclonal antibodies using *E. coli* as a host

Hybridoma technology is a kind of backbreaking, expensive, and long method. To beat these disadvantages, scientists attempt to create genetically changed microorganisms like bacteria to produce monoclonal antibodies. The aim is to develop bioreactors for the large-scale production of monoclonal antibodies in *E. coli* or in little microorganisms. The antigen-binding sites on the antibodies have a vital function within the binding of antibodies with the antigen; the Fv and Fab fragment play a major role. On the opposite hand, the Fc portion can be variable [42].

In this, the messenger RNA isolated from B-lymphocytes of humans or mice are converted into complementary DNA [cDNA]. The H and L chain sequences of this cDNA are amplified by using PCR. Then, these cDNAs are treated with restriction endonucleases to separate H and L chain sequences, and then these sequences are individually cloned into virus vectors. Later these are placed along and are cloned in another virus vector. The H and L chains get combined to create Fv fragments; then, these are screened for antigen-binding activity. The particular H and L chains forming of the inclusion are reworked into *E. coli* cells; then, this *E. coli* are cloned and propagated to provide Fv fragments that will bind to a particular antigen [42].

#### Site-directed Mutagenesis to produce monoclonal antibodies

**Fig. 7:**
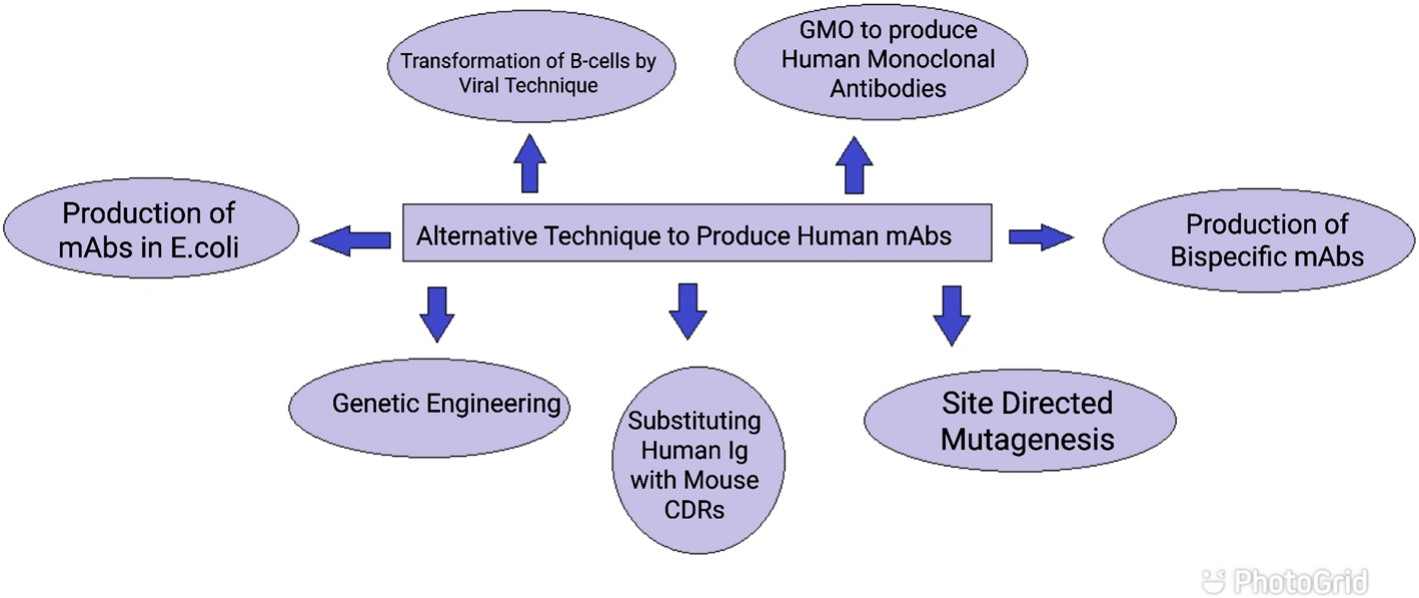
Techniques to produce human monoclonal antibodies

The site-directed mutagenesis could be a technique utilized in biotechnology; this method has created its potential to introduce amino acid residues at a very important position on the antibody. These amino acid residues can increase the potency of atom labeling and are established to be helpful in diagnostic imaging and radioimmunotherapy [41] (Fig. 7).

#### Anti-SARS-CoV-2 monoclonal antibodies

The spike (S), envelope (E), membrane (M), and nucleocapsid (N) structural proteins, as well as non-structural and auxiliary proteins, are all encoded by the SARS-CoV-2 genome. S1 and S2, two components of the spike protein, mediate host cell adhesion and invasion. In the treatment of SARS-CoV-2 infection, monoclonal antibodies that target the spike protein have been found to be effective [34]. The Food and Drug Administration (FDA) has issued Emergency Use Authorizations (EUAs) for three anti-SARS-CoV-2 monoclonal antibody products for the treatment of mild to moderate COVID-19 infection among non-hospitalized individuals with SARS-CoV-2 infection verified in the lab [43], such as:

- Bamlanivimab plus etesevimab: These are neutralizing monoclonal antibodies that bind to distinct but overlapping epitopes in the SARS-CoV-2 spike protein RBD [34, 43].
- Casirivimab plus imdevimab: It is human monoclonal antibodies that bind to non-overlapping epitopes of the SARS-CoV-2 spike protein, RBD. It is also used as a post-exposure prophylactic for those who are at high risk of contracting SARS-CoV-2 [34].
- Sotrovimab: This monoclonal antibody was discovered in a SARS-CoV survivor in 2003. It binds to an epitope in the spike protein’s RBD that is shared by SARS-CoV and SARS-CoV-2 [44].

However, anti-SARS-CoV-2 monoclonal antibodies are not currently approved for use in patients hospitalized with severe COVID-19; however, patients who have not developed an antibody response or who are not expected to mount an effective immune response to SARS-CoV-2 infection may be eligible for expanded access programs [44].

## Conclusion

The hybridoma technology uses the mature B lymphocytes present within the secondary lymphoid organ that are created in action against an invaded particular antigen. These B cells in the secondary lymphoid organ go through a maturation process in which the variable area of antibodies expands by collecting corporeal hypermutations, resulting in the selection of high-affinity binder antibodies. These antibodies have the ability to link substantial and lightweight chain genes in a natural way. In vivo and in vitro strategies are used for biological research and propagation functions [2, 11]. This method is suitable for the production of a huge number of monoclonal antibodies with specificity and selective binding for specific antigenic molecules like the external and internal structure of bacteria or virus, surface receptors of bacteria or virus, enzymes, chemicals, hormones, etc. Presently the majority of these antibodies are produced in mice or rabbits in the laboratory. These animals are available easily for immunization and are easy to control and handle in labs. Monoclonal antibodies are an excellent tool for scientific research and analysis and are widely used in various disciplines like biochemistry, immunology, molecular studies, pharmaceutical, and biotechnology [10] with a high degree of specificity, sensitivity, and reliability. These antibodies are used clinically; however, these antibodies carry some animal proteins that have a risk of inducement of immune responses in humans like hypersensitivity and allergies. However with expanded analysis scope in this field, numerous strategies are being developed to avoid these undesirable effects of the monoclonal antibodies [20]. There have been some attempts made to produce human monoclonal antibodies to reduce any undesirable side effects or attempts are made to make these antibodies as human as possible. The most economical and effective methodology of manufacturing antibodies would be to fuse antigen-specific human B-cells with human or mouse metastatic tumor cells. However, this methodology holds a drawback of manufacturing genetically unstable vegetative cells. Some other functions of monoclonal antibodies are these can be used to identify surface proteins or define surface antigen of a cell, and they can be also used to localize molecules within cells or tissues. The practical advantage of hybridoma technology is that after the stable somatic cell lines are established; then, these may be used to produce sensitive and specific monoclonal antibodies in limitless quantities. The antibodies created by the hybrid cells during this technology preserve the native paring of constant and variable regions of the antibodies. This helps within the analysis of the functioning of monoclonal antibodies. Hybridoma cell technology is one of the foremost vital technologies for the assembly of monoclonal antibodies [4]. Currently, the classic methodology of hybridoma cell technology is responsible for more than ninetieth of the antibodies licensed by the US Food and Drug Administration (US FDA) [10]. These monoclonal antibodies are utilized in chimeric or humanized versions, either directly or indirectly. With the recent development and research in this field of hybridoma technology, like the event of transgenic animals or the assembly of humanized antibodies, this technique has dominance over several alternative strategies used for the assembly of monoclonal antibodies [20]. In the present biotherapeutic age, researchers are working tirelessly to develop simpler and higher-quality human antibodies [5].

## Data Availability

Not applicable.

## Abbreviations

CDRs: Complementarity-determining regions
mAbs: Monoclonal antibody
HCG: Human chorionic gonadotropin
HGPRT: Hypoxanthine-guanine phosphoribosyl transferase
PEG: Polyethylene glycol
FDA: Food and Drug Administration
AIDS: Acquired immune deficiency syndrome
EBV: Epstein-Barr virus
HAT: Hypoxanthine-aminopterin-thymidine
HIV: Human immunodeficiency virus
